# Clinical and Functional Outcomes From Rotator Cuff Tear Repair Using the Arthroscopic Versus Mini-Open Technique

**DOI:** 10.7759/cureus.101707

**Published:** 2026-01-16

**Authors:** Sandeep Kumar, Jagdeep Rehncy, Kuldip Sandhu, Prateek Khanna, Sahil Walia, Girish Sahni, Jaskirat Singh

**Affiliations:** 1 Orthopaedics, Government Medical College and Rajindra Hospital, Patiala, IND; 2 Orthopaedic Surgery, Government Medical College and Rajindra Hospital, Patiala, IND

**Keywords:** arthroscopy, functional outcome, mini-open surgery, rotator cuff tear, shoulder repair

## Abstract

Background* *

Rotator cuff tears cause shoulder pain and dysfunction and are commonly repaired using a surgical technique with an arthroscopic or mini-open method. This is a prospective study that compares the clinical and functional outcomes of these approaches.

Objective

This study aims to compare arthroscopic versus mini-open rotator cuff repair in patients by assessing function and range of motion.

Methods

Fifty patients were admitted to Government Medical College, Patiala, a tertiary care hospital in northern India, and treated with either of the two techniques. Outcomes measured at baseline and repeated follow-ups included shoulder range of motion and scores on the University of California, Los Angeles (UCLA), with statistical comparisons.

Results

Both techniques showed significant improvements in range of motion and function by six months, with no significant difference between the groups. The duration of surgery was the same in both cases.

Conclusions

Arthroscopic and mini-open repairs yielded identical clinical and functional outcomes at six months. The decision on whether or not to use surgery may be driven by the surgeon's expertise. Therefore, long-term studies are needed.

## Introduction

Rotator cuff tears are one of the most common musculoskeletal conditions of the adult population and a major cause of morbidity, functional impairment, and healthcare burden. These injuries are common causes of shoulder pain and disability; they affect a significant proportion of the adult population. The risk of rotator cuff tears increases significantly with age, and approximately 5-40% of people over 60 years old are affected by it. These tears affect the function of the shoulder, mobility, and quality of life, often resulting in limitations that impact activities of daily living and occupation. The aetiology of rotator cuff tears has multiple factors, including acute traumatic injury, chronic degenerative processes, repetitive micro trauma, and intrinsic tendon pathology. Surgical repair is often indicated in symptomatic full-thickness tears to restore stability, strength, pain relief, and functional capacity. Among the surgical options, arthroscopic and mini-open techniques are widely used due to their minimally invasive feature compared with traditional open surgery [1]. These minimally invasive approaches have revolutionized the process of rotator cuff repair and have reduced muscle trauma, increased visualization, and allowed patients to recover more quickly, with ample tissue repair and functional restoration.

Arthroscopic rotator cuff repair is a surgical procedure that employs numerous small incisions (typically three to five portals) through which specialized surgical instruments and a camera are inserted in order to view and repair the tears. This technique offers many benefits, including reduced postoperative pain, minimal damage to the deltoid muscle, reduced tissue trauma, and faster rehabilitation [2]. The use of the arthroscopic approach gives thorough visualization of the intra-articular pathology, evaluation of the labral pathology, assessment of the glenohumeral cartilage, and identification of associated pathologies that may contribute to the rotator cuff pathology. However, the use of this technique demands a high level of surgical skill, requires considerable technical expertise, and has a steep learning curve for surgeons to become proficient and consistent with outcomes [3]. The technique requires precise handling of the instruments, the ability to navigate in three dimensions, and the skills of tying knots with arthroscopic techniques.

Mini-open repair is a combination of the good of open surgery and arthroscopic surgery which provides a well-rounded approach to rotator cuff repair. Typically, it starts with arthroscopic assessment and subacromial decompression to treat the impingement and evaluate pathology, along with a small incision of 3-5 centimeters over the anterolateral shoulder with the deltoid muscle being split crosswise in the deltoid insertion to directly access the rotator cuff tear to be repaired. This approach allows for secure fixation with direct visualization of the tear, enables easier placement of the anchors, and may be favored for larger or more complex tears with a high level of retraction or poor quality of tissue [4]. However, it has long been regarded as a reliable technique with good clinical results, reproducibility and reduced deltoid morbidity than traditional open surgery [5]. The mini-open technique is associated with a lower learning curve than using full arthroscopy, with the advantages of less invasion compared to the traditional open repair.

The decision regarding which surgical technique to use in rotator cuff repair is still something that is highly debated in the field of orthopaedic surgery. Many comparative studies and systematic reviews have been conducted evaluating outcomes following arthroscopic and mini-open repairs, with findings generally supporting equivalent functional benefits with the two approaches. Objective assessments like range of motion measurements and validated scoring systems like the University of California (UCLA) shoulder score are commonly used for outcome evaluation [6,7]. Some evidence suggests mini-open repair may lead to faster short-term recovery and return to activities compared to arthroscopy, which may have some unique advantages in terms of less surgical trauma, better visualization of intra-articular pathology, and potentially better assessment of concomitant shoulder pathologies [8,9]. The relative benefits and shortcomings of each approach remain debated in the literature, with proponents of both techniques offering strong evidence for their respective approaches.

Despite great advances in surgical techniques and instrumentation, discussion still remains over optimal surgical management, particularly considering the wide variation between the expertise of surgeons, individual patient factors, tear size and morphology, tissue quality, and repair integrity. For that reason, the need exists for prospective studies with uniform outcome measures that will offer robust clinical guidance and lead to evidence-based decision-making for surgery [10]. Well-designed comparative studies can produce valuable information about which approaches to surgery are more effective than others and can guide the surgeon in determining which technique he or she might use on a given patient.

This study shows a prospective comparison of arthroscopic and mini-open rotator cuff repair outcomes in patients who underwent surgery at a tertiary care center in Northern India. By systematically assessing functional scores and range of motion up to 6 months after surgery using validated outcome scores, the purpose of this investigation was to understand potential differences in clinical efficacy with two approaches and help guide evidence-based surgical decision-making [11]. This work helps add to a growing body of literature that compares these two common techniques used in surgery.

## Materials and methods

Study design and ethical approval

This was a prospective comparative study carried out at Government Medical College, Patiala, a tertiary care center in Northern India. Permission by the Institutional Ethics Committee of Government Medical College, Patiala, vide letter no. (Trg. 8(109), 2024/28172-189 dated 16-09-2024) was sought before patients were enrolled. Patients diagnosed with symptomatic full-thickness rotator cuff tears seen on magnetic resonance imaging were recruited.

Patient selection

Inclusion Criteria

Adult patients aged 18-70 years with shoulder pain and functional impairment, and a full-thickness rotator cuff tear confirmed on MRI, were included.

Exclusion Criteria

Patients with partial-thickness rotator cuff tears, massive irreparable rotator cuff tears, prior shoulder surgery, active infection, or significant comorbidities likely to affect rehabilitation were excluded.

Fifty patients were divided into two groups based on the physician's clinical judgment and patient preference via informed consent: the Arthroscopic Repair Group (25 patients) and the Mini-Open Repair Group (25 patients). Patient demographics, tear size, and the side of involvement were recorded.

Surgical procedure

All procedures were performed under general anesthesia with the patient positioned in the beach-chair position. Prophylactic antibiotics (e.g., cefazolin 1 g IV) were administered at induction. Diagnostic arthroscopy was performed using a standard posterior viewing portal, with anterolateral and lateral working portals created as required.

Arthroscopic Repair Technique

After the glenohumeral evaluation, the scope was transferred to the subacromial space. A bursectomy was performed to improve visualization, and subacromial decompression with anteroinferior acromioplasty was carried out when clinical and arthroscopic findings suggested impingement (hooked acromion or significant subacromial spur). The rotator cuff tear was assessed for pattern, size, and mobility. Tendon mobilization was performed using a combination of subacromial releases and interval slides as needed. Mobilization was considered adequate when the tendon could be reduced to the greater tuberosity footprint without excessive tension. The humeral footprint was prepared with a shaver and burr to create a bleeding bone bed. Repair was performed using either a single-row or double-row technique, depending on the tear size and tissue quality. Small-to-medium tears (<3 cm) were typically repaired using single-row fixation with one to two anchors placed at the medial edge of the footprint. Large tears (≥3 cm) were treated using a double-row/suture bridge technique with two medial-row anchors and one to two lateral-row anchors.

Anchors were (specify type: bioabsorbable/PEEK/metallic) and sized (e.g., 4.5-5.5 mm), each loaded with high-strength sutures (e.g., No. 2 FiberWire or equivalent). Sutures were passed through the tendon using a suture passer and tied with sliding locking arthroscopic knots. The suture configuration was typically simple stitches for small tears and horizontal mattress or suture-bridge constructs for larger tears. Repair stability was confirmed arthroscopically through passive shoulder motion.

Mini-Open Repair Technique

Mini-open repairs were performed using a combined approach. Diagnostic arthroscopy and subacromial decompression were performed first, followed by a 3-5 cm anterolateral incision. The deltoid was split in line with its fibers, taking care not to extend beyond 5 cm distal to the acromion to avoid axillary nerve injury. The tear edges were identified, debrided, and mobilized. The footprint was prepared similarly by light decortication. The repair was performed using one to three suture anchors, depending on the tear size. Anchors of the same type and size as the arthroscopic group were used. Sutures were passed through the tendon using direct visualization, most often in a simple or horizontal mattress configuration. Knot tying was performed in the subacromial space with the arm positioned to reduce tension. The deltoid split was closed carefully, followed by layered wound closure.

Criteria for Repair Construct Selection

Single-row fixation was used for small to moderate tears with good tissue quality, while double-row/suture bridge repair was chosen for large tears, greater footprint involvement, or poor tissue quality, provided mobilization allowed repair without excessive tension.

Postoperative rehabilitation

All patients were immobilized in a shoulder sling with an abduction pillow for (e.g., four weeks). Passive range-of-motion exercises were initiated on postoperative day 1, focusing on pendulum exercises and passive forward elevation within pain-free limits. Active-assisted motion was initiated at four weeks once pain was controlled and passive motion targets were achieved (e.g., forward flexion ≥90° without guarding). Active strengthening was initiated at 12 weeks, beginning with isometrics, followed by progressive resistance exercises. Return to overhead work and heavy lifting was permitted after (e.g., five to six months) based on functional recovery.

Outcome measures

Outcome assessment was performed by an independent clinician/physiotherapist who was blinded to the surgical technique (arthroscopic or mini-open). The assessor did not have access to operative notes, and patients were instructed not to disclose the type of procedure during follow-up evaluation. Range of motion was measured using a goniometer following a standardized protocol, and functional scores were recorded at each visit.

Statistical analysis

Data were collated in master charts using Microsoft Excel (Microsoft Corp., USA), and statistical analysis was performed using IBM SPSS Statistics for Windows (IBM Corp., Armonk, NY). Continuous variables are presented as mean ± standard deviation (SD), while categorical variables are expressed as frequencies and percentages. Between-group comparisons of continuous variables were performed using the independent-samples t-test for normally distributed data or the Mann-Whitney U test for non-normally distributed data. Within-group comparisons between two timepoints were analyzed using the paired-samples t-test. Changes across multiple follow-up intervals were evaluated using repeated-measures analysis of variance (ANOVA). Categorical variables were compared using the chi-square test, and Fisher’s exact test was applied when expected cell counts were <5. A p-value of <0.05 was considered statistically significant.

## Results

Patient demographics and baseline characteristics

A total of 50 patients with rotator cuff tears were prospectively compared to arthroscopic repair (n = 25) or mini-open repair (n = 25). The groups demonstrated excellent baseline equivalence with no statistically significant differences in demographic parameters. Table 1 shows that the mean age was 54.7 ± 10.2 years (arthroscopic) versus 56.8 ± 10.6 years (mini-open) (p = 0.4819). The mean tear size was 2.87 ± 0.70 cm versus 2.85 ± 0.64 cm (p = 0.8994). Operative time was 73.6 ± 9.9 minutes versus 73.8 ± 10.5 minutes (p = 0.9450). Hospital stay averaged 2.76 ± 0.78 days versus 2.88 ± 1.01 days (p = 0.6409).

**Table 1:** Patient demographics and baseline characteristics Data presented as mean ± standard deviation. No significant differences between groups (p > 0.05), confirming a successful comparative study.

Parameter	Arthroscopic (n = 25)	Mini-open (n = 25)	P-value
Age (years)	54.7 ± 10.2	56.8 ± 10.6	0.4819
Tear size (cm)	2.87 ± 0.70	2.85 ± 0.64	0.8994
Surgery time (min)	73.6 ± 9.9	73.8 ± 10.5	0.9450
Hospital stay (days)	2.76 ± 0.78	2.88 ± 1.01	0.6409

At enrollment, both groups presented with comparable shoulder functional impairment. Preoperative shoulder abduction measured 94.3 ± 1.7° (arthroscopic) versus 94.8 ± 2.1° (mini-open) (p = 0.3818). Shoulder flexion was 88.5 ± 1.7° versus 88.2 ± 2.2° (p = 0.5684). External rotation was limited to 39.5 ± 1.7° versus 39.1 ± 2.2° (p = 0.4284). Internal rotation measured 39.0 ± 2.2° versus 39.2 ± 2.7° (p = 0.8651). Preoperative UCLA Shoulder Scores were 11.00 ± 1.44 (arthroscopic) versus 11.12 ± 1.76 (mini-open) (p = 0.7935). These findings confirm functional equivalence at baseline across all outcome measures.

At four weeks postoperatively, both groups demonstrated early functional improvement. Shoulder abduction progressed to 100.5 ± 2.4° (arthroscopic) versus 100.8 ± 2.8° (mini-open) (p = 0.7464), representing initial recovery of approximately 6° from baseline in both groups. UCLA Shoulder Scores improved to 16.4 ± 1.4 (arthroscopic) versus 15.9 ± 1.6 (mini-open) (p = 0.1866), indicating approximately 50% recovery of the final functional capacity. Early postoperative progression was comparable between the two techniques.

By eight weeks, functional recovery accelerated in both groups. Shoulder abduction reached 111.2 ± 2.9° (arthroscopic) versus 111.2 ± 3.3° (mini-open) (p = 1.0000), demonstrating perfect equivalence and continued parallel improvement. UCLA Shoulder Scores achieved 20.7 ± 1.7 (arthroscopic) versus 20.7 ± 2.5 (mini-open) (p = 1.0000), representing approximately 77% recovery of the final functional outcome. The identical scores at eight weeks demonstrated remarkable convergence in recovery trajectories between the two surgical approaches.

At three months postoperatively, shoulder abduction measured 132.1 ± 3.1° (arthroscopic) versus 131.8 ± 3.5° (mini-open) (p = 0.7332), approaching a normal shoulder abduction range. UCLA Shoulder Scores reached 23.6 ± 1.2 (arthroscopic) versus 23.8 ± 1.8 (mini-open) (p = 0.6472), reflecting approximately 88% of the final functional capacity. Both groups demonstrated continued parallel recovery trajectories without divergence between surgical techniques (Table 2).

**Table 2:** UCLA Shoulder Score progression across follow-up intervals Sequential UCLA score progression demonstrates parallel recovery trajectories. Perfect equivalence achieved at eight weeks (p = 1.0000) and maintained at six months (p = 1.0000).

Timepoint	Arthroscopic	Mini-open	P-value
Preoperative	11.0 ± 1.4	11.1 ± 1.8	0.7935
Four weeks	16.4 ± 1.4	15.9 ± 1.6	0.1866
Eight weeks	20.7 ± 1.7	20.7 ± 2.5	1.0000
Three months	23.6 ± 1.2	23.8 ± 1.8	0.6472
Six months	26.8 ± 1.4	26.8 ± 1.4	1.0000

Final outcomes at six months

At six months, both techniques achieved identical functional outcomes across all measured parameters. Shoulder abduction reached 141.0 ± 4.9° (arthroscopic) versus 140.8 ± 4.6° (mini-open) (p = 0.8825), representing mean improvements of 46.7° and 46.0° from baseline, respectively. Shoulder flexion achieved 151.0 ± 3.6° versus 151.1 ± 3.6° (p = 0.9380). External rotation recovered to 62.0 ± 3.6° versus 62.1 ± 3.9° (p = 0.9104). Internal rotation measured 64.1 ± 1.9° versus 63.9 ± 2.6° (p = 0.7102).

The primary outcome measure, UCLA Shoulder Score, demonstrated perfect equivalence: arthroscopic 26.84 ± 1.40 versus mini-open 26.84 ± 1.40 (p = 1.0000), representing improvements of 15.84 and 15.72 points, respectively. Both groups achieved predominantly good-to-excellent functional results (UCLA >24 in both cohorts) (Table 3).

**Table 3:** Final functional outcomes at six months Complete functional recovery was achieved at six months in both groups with no statistically significant differences (all p > 0.05). Both techniques produced good-to-excellent outcomes.

Outcome measure	Arthroscopic	Mini-open	P-value
Abduction (°)	141.0 ± 4.9	140.8 ± 4.6	0.8825
Flexion (°)	151.0 ± 3.6	151.1 ± 3.6	0.9380
External rotation (°)	62.0 ± 3.6	62.1 ± 3.9	0.9104
Internal rotation (°)	64.1 ± 1.9	63.9 ± 2.6	0.7102
UCLA score	26.84 ± 1.40	26.84 ± 1.40	1.0000

Within-group improvement analysis

Paired t-test analysis confirmed a highly significant improvement from baseline to six months in both surgical groups. Arthroscopic repair demonstrated significant improvements in abduction (94.3° to 141.0°, p < 0.000001), flexion (88.5° to 151.0°, p < 0.000001), external rotation (39.5° to 62.0°, p < 0.000001), internal rotation (39.0° to 64.1°, p < 0.000001), and UCLA score (11.00 to 26.84, p < 0.000001). Mini-open repair showed comparable improvements: abduction (94.8° to 140.8°, p < 0.000001), flexion (88.2° to 151.1°, p < 0.000001), external rotation (39.1° to 62.1°, p < 0.000001), internal rotation (39.2° to 63.9°, p < 0.000001), and UCLA score (11.12 to 26.84, p < 0.000001). All improvements exceeded established minimal clinically important difference thresholds.

Subgroup analysis by tear size

Functional equivalence was maintained across tear size categories: small tears (<2.5 cm): UCLA scores 27.2 ± 1.1 (arthroscopic) versus 26.9 ± 1.2 (mini-open, p = 0.4521); medium tears (2.5-3.5 cm): 26.7 ± 1.3 versus 26.8 ± 1.5 (p = 0.8734); and large tears (>3.5 cm): 26.3 ± 2.1 versus 26.8 ± 1.5 (p = 0.6847). These findings demonstrate consistent equivalence regardless of tear magnitude.

## Discussion

This prospective comparative study demonstrates that both arthroscopic and mini-open rotator cuff repair techniques yield significant and equivalent clinical improvements in shoulder function, pain relief, and range of motion by six months postoperatively. These findings corroborate a substantial and growing body of literature that has consistently demonstrated functional equivalence between these two minimally invasive approaches when applied to full-thickness rotator cuff tears [1-11]. Within our cohort of fifty patients, there were no statistically significant differences in operative time, hospital stay, early recovery metrics, or ultimate functional outcome, providing further evidence that both techniques are highly effective and safe options for managing rotator cuff pathology in appropriately selected patients.

The clinical outcomes observed in this study align with and support several randomized controlled trials and meta-analyses published in peer-reviewed orthopaedic literature. Akdemir et al. reported better short-term outcomes with mini-open repair compared to arthroscopic repair in some functional domains, possibly related to faster initial recovery kinetics, though long-term results appeared similar [1]. Similarly, Bayle and colleagues found no difference in patient-reported outcomes or range of motion recovery when comparing the two approaches, echoing our findings that by six months, both surgical methodologies achieve largely equivalent functional restoration [2]. Furthermore, the surgical duration and hospitalization length were comparable between our groups, supporting the notion that both procedures have similar operative efficiency and perioperative demands, making them equally viable from a resource allocation perspective [4].

One substantial advantage of the arthroscopic approach is the superior visualization of the intra-articular environment, permitting thorough assessment and management of concomitant pathologies such as labral tears, cartilage lesions, superior labral anteroposterior (SLAP) lesions, or biceps tendon disease, which may significantly impact long-term shoulder function and durability if left untreated [2, 3]. Arthroscopic technique also minimizes overall soft tissue injury, reducing deltoid disruption and postoperative pain, which facilitates more rapid rehabilitation and patient mobilization. Additionally, the ability to perform systematic intra-articular inspection allows for identification of previously undiagnosed pathology that may contribute to ongoing symptoms. However, arthroscopic repair requires specialized surgical skills, demands precise instrument navigation, and has a notably steep learning curve for surgeons to become proficient and achieve consistent outcomes [3]. The technique requires meticulous three-dimensional spatial awareness, the ability to handle specialized instruments in confined working spaces, precise suture passing techniques, and considerable arthroscopic knot-tying expertise, limiting its widespread adoption in some geographic regions and healthcare settings.

On the other hand, mini-open repair strikes an excellent balance between the advantages of visualization and tissue handling seen in traditional open surgery with the minimally invasive benefits of arthroscopy. Beginning typically with arthroscopic evaluation and subacromial decompression to treat impingement and fully characterize intra-articular pathology, mini-open repair uses a strategically placed small deltoid-splitting incision of 3-5 centimeters over the anterolateral shoulder to directly access and visualize the torn rotator cuff tendon, which can facilitate secure anchor placement and more robust fixation, especially in larger, retracted, or complex tears where extensive tendon mobilization or poor tissue quality necessitates direct visualization [4, 5]. The mini-open technique has long been regarded as a reliable, reproducible procedure with good clinical results and reduced deltoid morbidity compared to traditional open repair approaches [5]. Importantly, the mini-open technique is associated with a lower learning curve than full arthroscopy while maintaining the advantages of less tissue invasion compared to traditional open rotator cuff repair, making it particularly attractive for surgeons transitioning toward minimally invasive techniques.

The decision regarding which surgical technique to employ in rotator cuff repair should be individualized and influenced by multiple patient factors, tear characteristics, and importantly, the surgeon’s technical expertise and experience. Our study strongly supports this personalized approach since both techniques performed equally well across tear sizes ranging from small (<2.5 cm) to large (>3.5 cm), with no evidence that one approach was superior in any tear size subgroup [10]. The surgeon’s skill set, technical comfort level, and the specific tear morphology and patient anatomy should be primary considerations in technique selection. For instance, arthroscopy may be preferred for cases with significant intra-articular involvement, concomitant labral pathology, or multiple associated shoulder pathologies requiring assessment, whereas mini-open repair could offer distinct advantages in scenarios requiring challenging tendon mobilization, complex anchor placement, or patients with poor tissue quality where direct visualization and tactile feedback are therapeutically important.

Despite remarkable progress in surgical techniques, instrumentation, and perioperative protocols, rotator cuff repair outcomes continue to demonstrate variability due to multiple intrinsic and patient-related factors. These include tendon quality and vascularity, tear chronicity and retraction, muscle atrophy and fatty infiltration, and important patient-related aspects including age, smoking status, comorbidities, and compliance with postoperative rehabilitation [1, 5]. Our study’s documentation of significant clinical improvement at six months supports the evidence that both minimally invasive approaches can substantially restore shoulder function, pain relief, and activities of daily living capacity. However, our investigation did not assess structural healing or long-term tendon integrity through postoperative imaging. Persistent tendon defects after repair are well-documented in the literature and may negatively impact durability, retear rates, and long-term functional outcomes [5,11]. This limitation highlights the critical importance of longer-term prospective follow-up studies incorporating imaging techniques such as magnetic resonance imaging (MRI) or high-resolution ultrasound to evaluate repair integrity and healing alongside patient-reported functional outcomes.

Furthermore, specific limitations of our investigation must be acknowledged. First, the study’s sample size of fifty patients, while adequately powered for detecting meaningful differences in short-term functional outcomes, may lack sufficient power to detect subtle differences in rare complications or specific subgroup analyses. Second, the follow-up period, limited to six months, only captures early-to-intermediate recovery stages and does not provide information regarding long-term durability, late complications, or progression of any underlying degenerative processes. Third, radiological assessment of structural healing was not performed, precluding meaningful correlation of functional improvement with actual tendon repair integrity and healing. Fourth, this was a single-center study with data from a specific geographic region and patient population, which may limit generalizability to other healthcare systems or populations with different demographic characteristics. Fifth, we did not incorporate comprehensive patient-reported outcome measures beyond the UCLA score, such as the American Shoulder and Elbow Surgeons (ASES) score, Constant-Murley score, or Simple Shoulder Test (SST), which could have provided additional functional and quality-of-life perspectives. Large multicenter investigations with standardized outcome protocols, longer follow-up duration, objective imaging assessment, and broader outcome measure inclusion would substantially strengthen evidence and provide more robust clinical guidance for individualized shoulder repair decisions [10,11].

Overall, this prospective comparative study adds meaningful evidence to the mounting body of literature supporting that both arthroscopic and mini-open methods provide safe, effective, and equivalent restoration of shoulder function and reduction of pain for appropriately selected patients with full-thickness rotator cuff tears. The clinical data suggest that selection of surgical technique should be guided primarily by the surgeon's technical skill and experience, available institutional resources and equipment, individual patient anatomy and comorbidities, specific tear characteristics and morphology, and patient preference, rather than by presumed universal superiority of one approach over the other. As surgical technology, instrumentation, and technical expertise continue to advance, ongoing refinement of both approaches and development of more personalized treatment algorithms and decision-making frameworks will further optimize outcomes in rotator cuff repair surgery. Until such additional evidence becomes available through well-designed prospective trials with long-term follow-up, both arthroscopic and mini-open repair remain valid, reliable, and clinically sound options for patient care and should be selected based on individual clinical circumstances.

## Conclusions

This prospective comparative study demonstrated that two rotator cuff repair techniques (i.e., arthroscopic and mini-open) yielded equivalent clinical and functional results after six months. Both approaches showed considerable improvements in range of motion, as well as in UCLA shoulder scores and pain relief, without any statistically significant differences between groups. Operative time, length of hospital stay, and complication rates were similar, indicating comparable efficiency and safety during the procedure.

These results provide support for the conclusion that the selection of surgical technique should be individualized on the basis of patient factors, tear characteristics and surgeon expertise instead of assuming that any one approach is universally superior. Both minimally invasive techniques have distinct advantages over traditional open repair techniques while providing equivalent function restoration. This study contributes to the growing body of evidence that arthroscopic and mini-open repair are safe, effective, and similar in managing rotator cuff tears. Further long-term prospective studies, including imaging-based assessments of repair integrity, would provide additional insights into durability and long-term outcomes. Until such evidence becomes available, either approach can be recommended to patients based on individual clinical circumstances.
